# Does Encouragement Matter in Improving Gender Imbalances in Technical Fields? Evidence from a Randomized Controlled Trial

**DOI:** 10.1371/journal.pone.0151714

**Published:** 2016-04-20

**Authors:** Cait Unkovic, Maya Sen, Kevin M. Quinn

**Affiliations:** 1 UC Berkeley School of Law, University of California, Berkeley, CA, United States of America; 2 John F. Kennedy School of Government, Harvard University, Cambridge, MA, United States of America; Northwestern University, UNITED STATES

## Abstract

Does encouragement help address gender imbalances in technical fields? We present the results of one of the first and largest randomized controlled trials on the topic. Using an applied statistics conference in the social sciences as our context, we randomly assigned half of a pool of 3,945 graduate students to receive two personalized emails encouraging them to apply (*n* = 1,976) and the other half to receive nothing (*n* = 1,969). We find a robust, positive effect associated with this simple intervention and suggestive evidence that women responded more strongly than men. However, we find that women’s conference acceptance rates are higher within the control group than in the treated group. This is not the case for men. The reason appears to be that female applicants in the treated group solicited supporting letters at lower rates. Our findings therefore suggest that “low dose” interventions may promote diversity in STEM fields but may also have the potential to expose underlying disparities when used alone or in a non-targeted way.

## Introduction

The scarcity of women in technical fields is well documented. According to the U.S. Department of Commerce, women hold close to half of all jobs in the United States, but just under a quarter of jobs in science, technology, engineering, and mathematics (STEM). Within engineering, the shortage is more acute, with women occupying around 14% of all jobs, a figure that has scarcely budged in ten years [1]. Despite these patterns, however, very few studies have used large-scale experimental methods to explore possible causal explanations behind them.

In this study, we apply techniques from large-scale social science interventions to gender imbalances in STEM. The experimental context is applications to a highly regarded applied statistics conference within political science, the Society for Political Methodology Summer Meeting (“PolMeth”). Our subject pool is 3,945 graduate students enrolled in the Top 50-ranked political science programs. From this subject pool, we randomly assigned half to receive two personalized emails encouraging them to apply to the conference (*n* = 1,976) and the other half to receive nothing (*n* = 1,969). This allows us to examine whether women (1) receive less information about opportunities within STEM fields or (2) receive less encouragement about STEM activities. To our knowledge, ours is among the first large-*N*, randomized controlled trials to explore either of these questions.

We examine two outcomes: (a) applications to the conference and (b) acceptance into the conference. With respect to the decision to apply, we find that those who received this simple treatment are more likely to apply than those who did not. This is the case even though our sample necessarily includes some individuals who would never apply to the conference (“never appliers”) under any circumstances. We also find strong suggestive evidence that women responded more strongly than men (although we are not able to rule out that there is no difference between the groups). When it comes to who was admitted to the conference, the picture is more mixed. Although we cannot estimate the average effect of encouragement on acceptance (conference slots are limited, and thus acceptance outcomes are not independent), we find that women’s conference acceptance rates are higher within the control group than in the treated group. This is not the case for men. Examining the applications further reveals the likely reason: women applicants in the treated group solicited a supporting letter of recommendation at much lower rates compared to the other groups. This suggests some caution. Although these kinds of “low-dose” interventions may be effective in increasing interest in STEM fields, such interventions have the potential to expose underlying disparities, particularly when they are used alone or in a non-targeted way. This raises additional questions for future researchers to examine.

This paper proceeds as follows. We first discuss the factors that previous research indicates are important contributors to the gender imbalance in STEM fields. We next describe our research design and present the core results documenting the baseline treatment effect. The remaining sections discuss faculty letters of support and results from a follow-up survey. We conclude with a discussion of the implications of this experiment, highlighting how this kind of research design can be fruitful for education policy researchers. Additional information, including examples of the interventions used, is provided in the Supporting Information.

## Possible Explanations Behind Gender Imbalances in STEM Fields

The existing literature highlights several factors that may play a role in the “leaky pipeline” of women’s involvement in STEM fields, though much of this evidence comes from small or exclusively observational studies. First, one set of studies finds that a possible explanation for gender imbalances in technical fields is the *relative scarcity of female role models*. For example, one observational study finds that having female instructors does indeed increase course enrollment and major selection in some STEM disciplines, but not in others [2]. This finding is consistent with other observational studies [3, 4], which find mostly positive relationships between the presence of female role models and female interest in technical fields. However, the finding is not consistent across all studies, as some find no relationship between the share of elite departments that are female and women’s choice of major [5].

Second, other survey-based studies have explored whether women’s *preferences over family, childbearing, and work-life balance* could contribute to the imbalance. For example, one study finds that female STEM graduate students are more likely than male students to express concern about the demands on current or future family obligations posed by the pursuit of prestigious scientific careers [6]. (Conditional on having neither children nor future plans to have children, the same study finds that attitudes between men and women hardly differ—speaking to the possibility of unmeasured confounders in such survey-based analyses.) Another study finds that marriage and family concerns are one of the most important factors dictating persistence in the STEM workforce [7]. More recently, survey-based work on academic career advancement suggests that this is an issue for women across all disciplines, not just those in technical areas [8]. That female role models in math and science may be more likely to be single or have no children makes these issues more salient in STEM fields.

Third, and related to the other phenomena, women are less likely to report favorably on *informal structures and networks* that would otherwise promote their research and intellectual development. For example, a 2010 report from the National Academy of Sciences notes that, “although women reported that they were more likely to have mentors than men, they were less likely to engage in conversations with their colleagues on a wide rage of professional topics, including research, salary, and benefits.” The report notes that this “distance may prevent women from accessing important information and may make them feel less included and more marginalized in their professional lives” ([9], p. 9). More informally, observations about the field we study here, applied statistics within political science, reflect a similar theme. According to one account:

In a subfield not famous for its practitioners’ social skills, male insecurity can lead to clumsy combative behavior that makes the atmosphere even colder. The cumulative effect can be depressingly powerful. One need not spend much time talking to women political scientists who have attended past [Political Methodology] Summer Methods meetings to hear dreadful stories of dismissive or belittling remarks ([10], p. 27).

These are observational studies, and most do not document all of the potential differences between male and female students. This reflects a broader hurdle for education policy research [11]. The problems with conducting large-scale experimental studies in an educational context are significant. First, randomizing treatments within a classroom makes it nearly impossible to avoid cross-contamination, and thereby SUTVA problems with causal analysis. Second, randomizing a treatment at the classroom (or department or university) level may result in very small *N*, which leads to studies that are underpowered for most treatment effects.

We note some experimental inroads, however, which motivate our approach here. One study asked panels of scientists to review identical vitas for a presumptive lab assistant position, one filed with a female name and the other with a male name; they find that CVs with female names are offered less in salary than those with male names, despite the CVs being otherwise identical [12]. In addition, researchers have estimated this bias using lab-based Implicit Association Tests, which suggest that the degree of bias against women is not only pervasive but predicts substantive differences in science and mathematics achievement [13]. For the most part, however, these experiments operate within highly controlled laboratory environments.

More directly related to our experiment are studies in behavioral economics that have used low-cost “nudge” interventions to surprising effect [14]. For example, randomized trials have found that texting students encouraging messages increases college attendance among low-income students [15], that mailing personalized informational packets about the college application process increases applications from low-income families [16], and that weekly text messages sent to high school students’ parents reduce the share of failing students [17]. As we show in the rest of this article, these sorts of experiments can be of great use to education policy research when used in a targeted way. These experiments also offer the rigor of a large-scale field design and the feasibility of easy-to-administer “low-dose” interventions. However, these studies for the most part focus on primary or high school education, rather than higher education. None of these studies, to our knowledge, have investigated the gender imbalance in STEM fields.

## Experimental Protocol

Thus, existing literature explores at least four interrelated mechanisms that may drive the under-representation of women in technical fields: (1) lack of role models and mentoring, (2) family and life balance concerns, (3) implicit or explicit bias, and (4) exclusion or professional/social ostracism. Our experiment touches upon two of these mechanisms, focusing specifically on the idea that women may systematically receive (i) less information about important professional and intellectual opportunities, and (ii) less encouragement. Indeed, that women receive less information and encouragement about engaging in STEM areas could flow directly from an unconscious bias by existing members of those communities; it could also flow from women being less integrated (both professionally and socially) in valuable networks that foster important intellectual and research opportunities.

Our experimental background is the Political Methodology Summer Conference (“PolMeth”), sponsored in part by the National Science Foundation. Hosted annually by a rotating cast of political science departments, the conference provides significant networking opportunities for graduate students and showcases new research on computer programming, machine learning, statistical theory, text analysis, data science, causal inference, and experimental methods. For these reasons, the political methodology subfield can be considered akin to more traditional STEM fields such as applied statistics, data science, or econometrics. In addition, gender imbalances within the political methodology community reflect broader gender imbalances within STEM. Roughly 25% of attendees at the annual PolMeth meeting are women [10, 18] and around 17% of co-authors or presenters are women. We also note that applied statistics is, like training in other STEM areas, increasingly important within the academic job market. In S1 Table, we present data showing that job market candidates who specialize in political methodology enjoy more favorable jobs-to-applicants ratios. PolMeth is therefore a good setting to understand the roots of gender imbalances in more technical fields.

Acceptance to the conference is competitive. The conference application requires students to submit a proposal and encourages them to solicit a faculty letter of support. Proposals are then subject to non-blind review by a committee of nine faculty members from across the community. Each year, approximately 70 to 80 slots are available for student presenters. Table 1 reports the number and gender of previous attendees and, to the extent available, applicants.

**Table 1:** Graduate Student Applications and Acceptances to PolMeth, 2010-2014.

	Applications			Acceptances		
Year	Male	Female	Gender Unknown	Male	Female	Gender Unknown
2010	–	–	–	39	21	14
2011	–	–	–	56	20	12
2012	–	–	–	44	19	4
2013	85	29	0	64	15	0
2014*	92	67	2	45	27	0

*Year of intervention

We conducted the intervention in the Spring of 2014. (The Harvard Committee on the Use of Human Subjects approved the intervention and follow-up survey described below, IRB Protocols #14-2683, #14-2864, and #16-0167. Due to its nature, we did not obtain consent prior to the intervention. Protocol #14-2683 approved this protocol. For the follow-up survey, we obtained survey respondents’ consent by asking them to indicate their consent before proceeding to the survey. Protocol #14-2864 approved this protocol. Protocol #16-0167 pertained to the continuation of the study.) To generate the potential subject pool of applicants, we collected the names and contact information of all graduate students in the Top 50 programs within political science. We first identified the 50 top-ranked programs, as determined by *U.S. News & World Report*. (Historically, the majority of conference applicants come from these programs.) We then searched each department’s webpage to determine the names and email addresses of enrolled graduate students. (Although some department webpages were out of date, to our knowledge this was random error with no systematic over- or under-reporting of names or email addresses according to gender or research area.) When possible, we denoted the gender of each student according to commonly used first names and/or online photographs. When gender could not be determined, it was denoted as unknown.

This search yielded 4,188 names and email addresses, including 2,478 men, 1,652 women, and 58 people of unknown gender across 53 departments. We subsetted these data to only those students with known gender and email address, producing an experimental subject pool of 3,945 students, 2,348 of whom are male and 1,597 of whom are female. We note that this pool of experimental subjects includes some students with little or no interest in statistics—for example, students pursuing a humanities oriented approach to political philosophy. Indeed, approximately 10–15% of all graduate students fall into this category [19], which makes them conference “never appliers” (i.e., people for whom the treatment effect would always be zero). As we discuss below, this makes our study different from *closely targeted* studies [16]. The primary consequence of this difference is that our treatment effect estimates are likely smaller than those that would have been obtained from an intervention on a more narrowly targeted population of potential applicants.

We then randomly assigned students to either a control or treated group. Because a student’s department is a predictor of conference attendance, and because the treatment effect could vary by gender, we blocked on both department and gender. In other words, for the fixed number, *n*_*dg*_, of students of gender *g* in department *d*, we randomly allocated half of the *n*_*dg*_ students to the treatment group and half to the control group. Where *n*_*dg*_ was not evenly divisible by two, we randomly allocated the one extra student to treatment or control with equal probability. We used a pseudo-random number generator in the R language for statistical computing to randomly assign students to the treatment and control groups. Blocking on factors (such as department and gender) that are thought to be predictive of outcomes is an accepted method of controlling experimental error and improving the precision of estimates ([20], Section 2.5). We randomly assigned treatment at the individual rather than group level in order to maximize statistical power. This could potentially lead to contamination across students (perhaps by sharing or forwarding the intervention email); we discuss this below.

Randomization produced 1,976 students assigned to treatment and 1,969 to control, bringing the total number of participants to *n* = 3,945. Of the 1,976 students assigned to treatment, 1,174 are men and 802 are women; 637 come from political science departments ranked in the Top 10, 611 from departments ranked 11 to 25, and 728 from departments ranked 26 to 50. Of the 1,969 students assigned to the control condition, 1,174 are men and 795 are women; 635 come from departments ranked in the Top 10, 610 from departments ranked 11 to 25, and 724 from departments ranked 26 to 50. The Supporting Information includes gender breakdown by department and additional summary statistics (see S3 and S4 Tables).

The intervention came in the form of two encouraging emails, one on March 4, 2014, and the other on March 19, 2014. We sent both emails from the personal account of the current president of the Society for Political Methodology, the academic organization hosting the conference, and used the student’s first name for personalized encouragement. The emails, which we include along with selected responses in the Supporting Information (S1 and S2 Texts), discussed some of the benefits of attending the conference and concluded by encouraging the student to consider applying. General calls for proposals began around March 3, 2014, approximately one day before the first email intervention. These general calls included emails to various lists, including the PolMeth listserv, which means that some portion of students likely received both the experimental interventions and other promotional notices. We have no reason to believe that the promotional emails affected either the control or treated groups disproportionately.

Soon after the deadline for applications (March 28, 2014), we collected from the conference organizers (1) the names and affiliations of all graduate students who applied to the conference, (2) the names and affiliations of sponsoring faculty (if any), (3) proposal titles and abstracts, and (4) acceptance status. We combined these data with the data on whether the students had or had not received the treatment.

## Results

We present results relating to two outcomes: (1) a student’s decision to apply to the conference, and (2) whether the student’s proposal was accepted.

### Analysis of Applications to the Conference

We first examine the treatment effect on students’ decisions to apply to the conference. Table 2 presents the raw number of applicants by treatment status, broken down by sex and tier of school. Table 3 presents estimates of the sample average treatment effect (SATE), which in this case is the fraction of applicants among the treated students minus the fraction of applicants among the control students, averaged over gender and tier of school. This table also presents randomization-based *p*-values for the null hypothesis of no individual-level effect.

**Table 2:** Summary of Applications by Treatment Status and Subgroup.

Subgroup	*n*	*n* Treated	*n* Control	Treated Applicants	Control Applicants
Full Sample	3945	1975	1970	95	42
Men	2348	1173	1175	54	27
Women	1597	802	795	41	15
Top 10	1272	636	636	50	26
Men Top 10	758	378	380	31	18
Women Top 10	514	258	256	19	8
Top 11 to 25	1221	611	610	23	12
Men Top 11 to 25	734	367	367	12	7
Women Top 11 to 25	487	244	243	11	5
Top 26 to 50	1452	728	724	22	4
Men Top 26 to 50	856	428	428	11	2
Women Top 26 to 50	596	300	296	11	2

**Table 3:** Sample Average Treatment Effects of Encouragement on Application.

Subgroup	*n*	SATE Estimate	*p*-value
Full Sample	3945	0.027	<0.001
Men	2348	0.023	0.002
Women	1597	0.032	<0.001
Top 10	1272	0.038	0.003
Men Top 10	758	0.035	0.042
Women Top 10	514	0.043	0.035
Top 11 to 25	1221	0.018	0.081
Men Top 11 to 25	734	0.013	0.317
Women Top 11 to 25	487	0.024	0.204
Top 26 to 50	1452	0.025	<0.001
Men Top 26 to 50	856	0.021	0.013
Women Top 26 to 50	596	0.030	0.018

Table 3 shows positive, statistically significant effects of encouragement on the decision to apply for students overall and for students in all subgroups except those from schools ranked 11th to 25th. The overall effect of the encouragement is to increase applications by about 2.7 percentage points. Applications from men increase by about 2.3 percentage points, and applications from women increase by about 3.2 percentage points. The treatment effects are largest for students from Top 10 schools (a 3.8 percentage point increase compared to 1.8 percentage points and 2.5 percentage points for the other tiers). These schools, on average, have the strongest graduate training in statistics, suggesting that our encouragement is most likely to influence students who self-select into graduate programs known for quantitative research.

Is there any evidence that treatment effects are larger for women than for men? We adopt a Bayesian approach due to the small number of students in some gender-school strata and the relative ease with which uncertainty statements can be constructed regarding differences in SATE. (Our results do not hinge on the particulars of this approach. A reasonable frequentist approach yields qualitatively similar results.) For purposes of this analysis, we assume that application decisions within each university-gender stratum are generated from a binomial distribution with a university-gender-specific probability of application. We take the prior distribution for each of these parameters to be a beta distribution with the first parameter equal to 0.1 and the second parameter equal to 0.1. As shown in Table 4, these Bayesian estimates of SATE are identical to the standard estimates in Table 3. The quantity labeled *δ* in Table 4 is the SATE for women minus the SATE for men. A positive value of *δ* implies that the intervention created a greater increase (in percentage point terms) for women than for men. While the point estimates of *δ* are positive—for the full sample and for all three tiers of schools—the 95% credible intervals include 0. Examining the posterior probability that *δ* > 0, we see suggestive, but not conclusive, evidence that *δ* is positive. The strongest evidence comes from the full sample, where we calculate that the posterior probability that *δ* > 0 is about 0.87.

**Table 4:** Female—Male Heterogeneity in Sample Average Treatment Effects of Encouragement on Application.

	Male	Female		95% CI for *δ*		
Subgroup	SATE	SATE	*δ*	lower	upper	Pr(*δ* > 0)
Full Sample	0.023	0.032	0.009	-0.007	0.026	0.869
Top 10	0.035	0.043	0.008	-0.028	0.044	0.669
Top 11 to 25	0.013	0.024	0.011	-0.016	0.039	0.793
Top 26 to 50	0.021	0.030	0.009	-0.013	0.031	0.794

Because most graduate programs do not list students by research area, our subject pool includes students whose interests do not include data analysis. Such students are unlikely to apply to an applied statistics conference regardless of encouragement. As we noted above, data from the American Political Science Association suggest that approximately 10–15% of all graduate students are involved in the study of political philosophy [19]. We can calculate a back-of-the-envelope revised SATE conditional on “possible appliers,” relying on the fact that the total SATE is the weighted sum of the conditional sample average treatment effect among the “never appliers” (NA) and the conditional sample average treatment effect among the “possible appliers.” In notation:

$$\begin{array}{ccc} SATE & = & \frac{1}{\left|I\right|}\sum_{i\in I}\left[Y_{i}\left(1\right)-Y_{i}\left(0\right)\right]\\ & = & \frac{1}{\left|I\right|}\left[\sum_{i^{\prime }\in I_{na}}\left[Y_{i^{\prime }}\left(1\right)-Y_{i^{\prime }}\left(0\right)\right]+\sum_{i^{\prime \prime }\in I_{a}}\left[Y_{i^{\prime \prime }}\left(1\right)-Y_{i^{\prime \prime }}\left(0\right)\right]\right]\\ & = & \frac{1}{\left|I\right|}\left[0+\sum_{i^{\prime \prime }\in I_{a}}\left[Y_{i^{\prime \prime }}\left(1\right)-Y_{i^{\prime \prime }}\left(0\right)\right]\right]\\ & = & \frac{1}{\left|I\right|}\sum_{i^{\prime \prime }\in I_{a}}\left[Y_{i^{\prime \prime }}\left(1\right)-Y_{i^{\prime \prime }}\left(0\right)\right] \end{array}$$ (1)

where I is the set of all experimental subjects, $I_{na}$ is the set “never appliers,” and $I_{a}$ is the set of “possible appliers.” The SATE among the “possible appliers” is

$$\begin{array}{c} SATE_{a}=\frac{1}{|I_{a}|}\sum_{i^{\prime \prime }\in I_{a}}\left[Y_{i^{\prime \prime }}\left(1\right)-Y_{i^{\prime \prime }}\left(0\right)\right]. \end{array}$$ (2)

*SATE*_*a*_ is thus $\left|I|/\right|I_{a}|$ times larger in magnitude than the overall *SATE*.

Using the more conservative 10% “never applier” rate gives us a revised SATE of around 3 percentage points overall, and around 3.6 percentage points for women. Using a less conservative 15% “never applier” rate gives us a revised SATE of around 3.17 percentage points overall, and around 3.76 percentage points for women. Thus, we have reason to think that a more closely targeted study—one that only targets those people whom policy experts have an *a priori* reason to think might respond—will actually achieve greater treatment effects. Here, taking into account this “never applier” phenomenon increases the treatment effect by roughly 10%.

### Analysis of Acceptances Into the Conference

We next investigate the relationship between treatment status and application success. We note that the conference committee was tasked with accepting a fixed number of applicants and could not increase or decrease that number. This creates conceptual problems for standard definitions of causal effects, and for the requirement that treatment assignment be independent of potential outcomes [21]. We therefore present descriptive statistics on this question in Table 5. The first column reports the number of students in each subgroup. The second and third columns report the fraction of accepted students within each subgroup disaggregated by treatment status. The denominator is all students in the relevant subgroup—both those who applied (and thus could have been accepted) and those who did not apply (and thus could not have been accepted). The final column reports the randomization *p*-value for a test of no individual-level effect whatsoever. That is, the null hypothesis here is that no student’s acceptance status would have been changed by changing his or her treatment status.

**Table 5:** Comparison of Acceptance Rates by Treatment Status (All Students).

Subgroup	*n*	Fraction Accepted among Treated	Fraction Accepted among Control	*p*-value
Full Sample	3945	0.020	0.013	0.076
Men	2348	0.022	0.013	0.093
Women	1597	0.016	0.013	0.533
Top 10	1272	0.038	0.022	0.083
Men Top 10	758	0.042	0.021	0.077
Women Top 10	514	0.031	0.023	0.746
Top 11 to 25	1221	0.015	0.015	0.841
Men Top 11 to 25	734	0.016	0.016	0.851
Women Top 11 to 25	487	0.012	0.012	0.751
Top 26 to 50	1452	0.008	0.003	0.167
Men Top 26 to 50	856	0.009	0.002	0.338
Women Top 26 to 50	596	0.007	0.003	0.611

From Table 5, we see higher acceptance rates for the treated students than for the control students, with the exception of students from departments ranked 11 to 25. There were no differences at all for this latter group of students. While we cannot reject the null hypothesis of no individual-level effect whatsoever for any subgroup at the 0.05 level, we can reject this null at the 0.10 level for the full sample, all male students, all students from top 10 schools, and male students from top 10 schools. We cannot reject the null hypothesis for any subgroup of women at any reasonable significance level. We therefore have suggestive evidence that treated students were more likely to be accepted to the conference than control students. Male students, however, appear to drive this difference.

Examining the fraction of accepted students within the control group reveals additional patterns. Here we see no major differences in acceptance rates by gender either in the full sample or within tier of school. A simple calculation ignoring statistical uncertainty suggests that, in a counterfactual world in which the outreach experiment was not conducted, the ratio of men to women accepted to attend the conference would have been about 1.47 to 1. This can be compared to the actual male-to-female ratio of 1.67 to 1 (see Table 1). Thus, we have weak evidence that the outreach experiment may have actually *worsened* the final female-male gender balance at the conference.

Because the groups of treated and control students who applied may not be balanced due to the treatment intervention being randomly assigned prior to the submission of applications, we direct our attention to those students who applied to attend the conference by examining descriptive data, presented in Table 6. (The total number of applicants in this table (137) does not equal the number of total applicants in Table 1 (161). This is the result of 24 applicants from non-U.S. institutions and non-top-50 U.S. institutions.) The first column of this table reports the number of students in each subgroup. The second and third columns report the fraction of accepted students within each subgroup disaggregated by treatment status. The final column reports the randomization *p*-value for a test that we describe below.

**Table 6:** Comparison of Acceptance Rates Among Applicants by Treatment Status.

Applicant Subgroup	*n*	Fraction Accepted among Treated	Fraction Accepted among Control	*p*-value
Full Sample	137	0.411	0.595	0.046
Men	81	0.481	0.556	0.538
Women	56	0.317	0.667	0.015
Top 10	76	0.480	0.538	0.617
Men Top 10	49	0.516	0.444	0.771
Women Top 10	27	0.421	0.750	0.206
Top 11 to 25	35	0.391	0.750	0.026
Men Top 11 to 25	19	0.500	0.857	0.166
Women Top 11 to 25	16	0.273	0.600	0.286
Top 26 to 50	26	0.273	0.500	0.358
Men Top 26 to 50	13	0.364	0.500	1.000
Women Top 26 to 50	13	0.182	0.500	0.415

The differences in acceptance rates in this table should not be interpreted causally. Not only is there the same concern about interference among units that we discussed regarding Table 5, but conditioning on a post-treatment variable threatens the independence of treatment status and unmeasured confounding variables. However, we can use the data in Table 6 to infer whether the program committee selected participants from among the applicants in a way that was statistically independent of treatment status. The program committee was not given any information on the treatment status of any individual and most of the committee members were not aware of the experiment. Thus, a rejection of the null hypothesis of independence of acceptance decisions and treatment status would suggest that unmeasured background factors that entered into the committee members’ decision making processes differ systematically between the control applicants and the treated applicants.

The null hypothesis we employ here assumes that the number of acceptances is fixed within each gender-tier combination and that these acceptances are randomly assigned to applicants within the relevant gender-tier stratum independently of treatment status. (It is also possible to think of the null as fixing the total number of acceptances overall but putting no constraints on the number of acceptances within gender-tier strata. Results based on this null hypothesis are qualitatively similar to those reported in Table 6, albeit with *p*-values that are slightly larger. In particular, the *p*-value for the full sample becomes 0.066, the *p*-value for all women becomes 0.030, and the *p*-value for all top 11 to 25 students becomes 0.067.) The *p*-values in Table 6 are randomization *p*-values that compare the observed difference in acceptance rates to the differences that arise from the appropriate randomization distribution.

Table 6 shows that we can reject the null of independence at the 0.05 level for the full sample, for all women, and for students from programs ranked 11 to 25. In each case, the fraction of accepted applicants is higher among the control applicants than among the treated applicants. We are not able to reject the null of independence for any of the male-only subgroups. Indeed, in one of the male subgroups (men at top 10 departments), the fraction of accepted applicants is higher among the treated applicants than among the control applicants, although not significantly so.

These results suggest that some aspects of the applications submitted by the treated women tend to differ from the applications submitted by the control women, and that these unmeasured differences in applications are associated with higher acceptance rates for the control women. Numerous self-selection stories are possible.

## Follow-Up Survey

After the conference, we contacted all of the 3,945 students in the study by email, requesting that they participate in an Internet-based follow up survey. Of those contacted, 1,629 (41%) students responded to at least one of the survey questions. Of these, 786 (48%) were treated students and the remaining 843 (52%) were students in the control group.

The survey asked questions regarding each student’s demographics, academic background, and experience in graduate school. The survey also asked the treated students whether and to what extent they forwarded the encouragement email to other students. These questions addressed the potential for spillover or contamination effects. Results from the follow-up survey suggest that this is not a serious concern. Of the 786 treated students, 22 (3%) reported that they forwarded the encouragement email to other students in their department and 8 of the 786 (1%) reported that they forwarded the encouragement email to students outside their department. Only 3 (0.003%) treated students reported that they forwarded the email to an institutional email list. This suggests that any spillover or contamination effects were likely minimal.

The follow-up survey allows us to examine possible reasons for the differences in acceptance rates in Table 6. One possibility is that the treated women tended to be less objectively qualified—perhaps because they were earlier in their graduate careers and/or had taken fewer quantitative methods courses. One question on the follow-up survey asked respondents how many years they have been in their current graduate program. We obtained responses for this question from 26 of the treated female applicants and 10 of the control female applicants. While the mean number of years is slightly lower for the treated women than for the control women (4.3 to 4.6), a two-sample *t*-test of the difference is not significant at conventional levels (*p*-value = 0.57). Neither do we see major differences in the number of quantitative methods classes taken. All respondents report having taken 5 or more classes with the exception of one treated female applicant and one control female applicant who each reported taking 4 quantitative methods courses.

An attribute that does seem to vary systematically by treatment status of female applicants who responded to the survey is their stated area of study. Table 7 presents these data. Treated female applicants are more likely to work in the areas of comparative politics and international relations than are their control counterparts. These students constitute the bulk of the rejections among the treated female applicants. Interestingly, the only female applicants who listed quantitative methods as their main field were in the treated group. Two of these three students were rejected from the conference.

**Table 7:** Subfields of Female Applicants (Among Survey Respondents).

Subfield	Treated Female Applicants	Control Female Applicants	Rejected Treated Female Applicants	Rejected Control Female Applicants
American	5	5	1	3
Comparative	13	4	9	0
IR	4	1	3	0
Methodology	3	0	2	0

## Letters of Support

The conference data also reported (1) whether a letter of support was submitted for the applicant and, if so, (2) which faculty member wrote it. We have these data for all 56 of the female applicants who were in the outreach experiment. Table 8 reports whether a student’s recommender submitted a letter of recommendation before the deadline and whether that letter was from a “networked” or “non-networked” advisor. We define a “networked” advisor to be someone who is a) a fellow of the Society for Political Methodology, the academic society sponsoring the conference, b) a winner of a Society-sponsored award, c) a current or former officer of the Society, or d) a current or former member of a Society committee.

**Table 8:** Letters of Recommendation of Female Applicants (Among All Female Applicants).

Letter of Rec.	Treated Female	Control Female	Rejected Treated Female	Rejected Control Female
Networked Letter	10	6	3	0
Non-Networked Letter	10	5	5	1
No Letter	21	4	20	4

Table 8 shows that *the major difference between the treated and control female applicants is whether the applicant’s advisor submitted a letter before the application deadline*. About 51% of the treated female applicants were lacking a letter of recommendation compared to 27% of the control female applicants. While the lack of a letter of recommendation was not formally disqualifying, only 1 of the 25 female applicants without a letter was accepted to the conference. This single factor seems to explain much of the difference in acceptance rates between treated and control women. Among female applicants with letters of recommendation, we do not see a major difference in the percentage of networked versus non-networked letters. Table 9 presents equivalent data for male applicants.

**Table 9:** Letters of Recommendation of Male Applicants (Among All Male Applicants).

Letter of Rec.	Treated Male	Control Male	Rejected Treated Male	Rejected Control Male
Networked Letter	22	10	4	1
Non-Networked Letter	9	6	3	2
No Letter	23	11	21	9

## Discussion and Conclusion

To summarize our contributions, we find that a simple email intervention increased interest in a STEM-related conference. We also provide suggestive, though not fully conclusive, evidence that the encouragement had a stronger effect among female students. This is a straightforward, low-cost intervention, and one that can be applied across other STEM areas and perhaps generate more interest in the more technical areas of the social sciences. In addition, as we noted above, the treatment effect associated with such interventions could be substantially strengthened if combined with close targeting of the population.

However, our findings also suggest that such simple, large-scale interventions have the potential to expose other problems. In our experiment, the encouragement led to increased applications among the treated female graduate students, but these new applicants failed to gain acceptance into the PolMeth conference at rates equal to either the male applicants or the female applicants in the control condition. Although other research has raised the possibility of implicit bias against female STEM participants [12], which could possibly apply in the application-review stage here, we believe that a more compelling explanation is that female students in the treated condition were more likely to apply to the conference without having procured a faculty letter of support. Thus, the female treated students appeared to have weaknesses in their applications that may have translated into increased rejections. This is consistent with previous research demonstrating that female STEM students may be more likely than male students to lack mentoring and networking opportunities [9], which in turn is consistent with the differences in “networked” letters of support by gender seen in Tables 8 and 9. This pattern is also consistent with research showing that male students develop greater professional confidence than do female students [22], a factor that perhaps emboldens more male students to ask for letters of support. Finally, we note that this pattern may also be consistent with students who received the encouragement feeling that they would be automatically accepted to the conference (or receive some type of preferential treatment in the selection), leading them to put less effort into the application process and to be less likely to procure a supporting letter. (We note, however, that this last explanation does not fully account for the observed differences in the treated male and female students in terms of faculty letters.) Our findings ultimately suggest that, although encouragement can be effective in engaging female students, it may intensify pre-existing imbalances—and in the process may do a disservice to groups historically marginalized in more technical fields. Increasing interest alone may not be sufficient to overcome more serious or more longstanding obstacles to participation.

We see several avenues for future research. First, future studies may explore whether such simple encouragement could also be counter-productive over longer periods of time. If encouraged female students apply to STEM-related programs but are rejected at higher rates, these negative outcomes could actually serve to suppress potential future interest. More optimistically, however, the email intervention here was extremely simple and provided no additional information about either letters of support, proposal preparation, or other specific (or tailored) instructions. Future research may consider whether more detailed interventions—ones that provide additional offers of assistance, reminders about deadlines, and inside information about what makes a competitive proposal—would lead to different and improved results among female subjects.

More broadly, our contributions here also highlight that modern social science methods—including large-sale experimental techniques—can be implemented in education policy more generally. Here, the interventions of interest were two personalized emails. Education policy researchers can examine the effects of these sorts of individual-level interventions with similar large-scale field experiments, doing so not just at the primary or high-school level, but also in professional or doctorate education. Indeed, we hope that ours will be among many such studies coupling advances in “big data” with rigorous causal inference techniques to help answer broad questions in education policy.

## Supporting Information

S1 Table Importance of Quantitative Methods on the Job Market. Data on the importance of research expertise in quantitative methods for the academic job market in political science, as reported by the American Political Science Association.
(PDF)

S2 Table Political Methodology Attendance. Data on the number of attendees registered to attend the PolMeth conference (across both faculty and graduate students) and the share who are women, from 1984 (the first year of the conference) to the present. Since the mid-to-late 1990s, women have comprised about 20 to 25% of the attendees at the conference.
(PDF)

S3 Table Experimental Subjects by Department. Data on the number of male and female graduate students in the study, disaggregated by home department.
(PDF)

S4 Table Treated/Control Subjects by Department. Data on the treatment status of students, disaggregated by home department and gender.
(PDF)

S1 Text Intervention Email Text. Text of the two emails used as the randomized interventions.
(PDF)

S2 Text Responses to the Email Intervention. Examples of responses received to the two randomized interventions.
(PDF)
