# Supplementary material for: Does Encouragement Matter in Improving Gender Imbalances in Technical Fields? Evidence from a Randomized Controlled Trial
Source: PLoS One. 2016 Apr 20;11(4):e0151714. doi: 10.1371/journal.pone.0151714 (PMC4838300; doi:10.1371/journal.pone.0151714)
Supplement: S1 Table — Data on the importance of research expertise in quantitative methods for the academic job market in political science, as reported by the American Political Science Association. (PDF) [file pone.0151714.s001.pdf]

Importance of Quantitative Methods in the Job Market

	AY 2009-10		
Area	Applicants	Jobs	Applicants/Job
American Politics	178	105	1.70
International Relations	219	96	2.28
Comparative Politics	290	85	3.41
Political Philosophy	141	26	5.42
Quantitative Methods	16	4	4

	AY 2010-11		
Area	Applicants	Jobs	Applicants/Job
American Politics	214	120	1.78
International Relations	225	123	1.83
Comparative Politics	341	94	3.63
Political Philosophy	113	34	3.32
Quantitative Methods	4	10	0.4

	AY 2011-12		
Area	Applicants	Jobs	Applicants/Job
American Politics	210	122	1.72
International Relations	281	147	1.91
Comparative Politics	276	100	2.76
Political Philosophy	130	32	4.06
Quantitative Methods	9	9	1

Table S1. Number of candidates and tenure-track job posts (at Assistant Professor rank) by research area, AYs 2009-10, 2010-11, 2011-12. Although the number of jobs specifically calling for methodology is smaller than others, we note that nearly all of the job postings in American politics call for some expertise in quantitative methods, as do many jobs in comparative politics and international relations (indicating quantitative methods as a secondary specialty). As a result, the number of open positions per area alone understates the degree to which familiarity with statistical methods matters in procuring a position. Source: American Political Science Association.
