# Supplementary material for: Does Encouragement Matter in Improving Gender Imbalances in Technical Fields? Evidence from a Randomized Controlled Trial
Source: PLoS One. 2016 Apr 20;11(4):e0151714. doi: 10.1371/journal.pone.0151714 (PMC4838300; doi:10.1371/journal.pone.0151714)
Supplement: S2 Table — Data on the number of attendees registered to attend the PolMeth conference (across both faculty and graduate students) and the share who are women, from 1984 (the first year of the conference) to the present. Since the mid-to-late 1990s, women have comprised about 20 to 25% of the attendees at the conference. (PDF) [file pone.0151714.s002.pdf]

Political Methodology Attendance

Year	Males	Females	Total	%Males	%Females
1984	15	1	16	93.8%	6.3%
1985	18	0	18	100.0%	0.0%
1986	18	1	19	94.7%	5.3%
1987	16	1	17	94.1%	5.9%
1988	14	0	14	100.0%	0.0%
1989	17	1	18	94.4%	5.6%
1990	28	9	37	75.7%	24.3%
1991	53	6	59	89.8%	10.2%
1992	49	10	59	83.1%	16.9%
1993	55	12	67	82.1%	17.9%
1994	41	9	50	82.0%	18.0%
1995	44	8	52	84.6%	15.4%
1996	51	9	60	85.0%	15.0%
1997	57	15	72	79.2%	20.8%
1998	87	19	106	82.1%	17.9%
1999	88	29	117	75.2%	24.8%
2000	96	22	118	81.4%	18.6%
2001	87	20	107	81.3%	18.7%
2002	92	23	115	80.0%	20.0%
2003	91	13	104	87.5%	12.5%
2004	106	27	133	79.7%	20.3%
2005	122	32	154	79.2%	20.8%
2006	70	25	95	73.7%	26.3%
2007	135	36	171	78.9%	21.1%
2008	181	59	240	75.4%	24.6%
2009	139	37	176	79.0%	21.0%
2010	129	43	172	75.0%	25.0%
2011	139	32	171	81.3%	18.7%
2012	123	35	158	77.8%	22.2%
2013	123	32	155	79.4%	20.6%
2014	109	39	148	73.6%	26.4%

Table S2. Historical PolMeth Attendance (Faculty and Student) by Sex. Source: Society for Political Methodology.
