# Supplementary material for: Does Encouragement Matter in Improving Gender Imbalances in Technical Fields? Evidence from a Randomized Controlled Trial
Source: PLoS One. 2016 Apr 20;11(4):e0151714. doi: 10.1371/journal.pone.0151714 (PMC4838300; doi:10.1371/journal.pone.0151714)
Supplement: S3 Table — Data on the number of male and female graduate students in the study, disaggregated by home department. (PDF) [file pone.0151714.s003.pdf]

Experimental Subjects by Department

University	Female	Male	Dept. Rank
Harvard	68	101	1
Princeton	39	96	2
Stanford	48	45	3
University of Michigan	37	40	4
Yale University	52	66	4
University of California, Berkeley	53	76	6
Columbia University	77	99	7
MIT	32	48	8
University of California, San Diego	31	75	8
Duke	28	44	10
University of California, Los Angeles	49	68	10
University of Chicago	60	75	12
University of North Carolina, Chapel Hill	35	45	13
Washington University in St. Louis	12	24	13
New York University	27	55	15
Ohio State University	27	49	15
University of Rochester	15	34	15
University of Wisconsin-Madison	32	37	15
Cornell University	41	50	19
University of Minnesota, Twin Cities	34	48	19
Northwestern University	46	55	21
The University of Texas at Austin	45	77	21
University of California, Davis	19	42	23
University of Illinois at Urbana-Champaign	26	33	23
Emory University	19	18	25
Indiana University Bloomington	36	73	25
Texas A&M University, College Station	13	19	25
Penn State University	16	30	28
University of Maryland	19	27	28
University of Pennsylvania	24	36	28
University of Washington	48	43	28
Michigan State University	16	34	32
Rice	7	23	32
Stony Brook University	13	14	32
The University of Iowa	15	22	32
Notre Dame	34	53	36
The George Washington University	24	40	36
University of Virginia	29	39	36
Vanderbilt University	21	25	36
Florida State University	14	27	40
Georgetown University	58	62	40
Johns Hopkins University	5	7	40
University of California, Irvine	29	44	40
University of Pittsburgh	15	25	40
Brown University	24	29	45
Rutgers	52	40	45
University of Colorado, Boulder	25	39	45
University of Arizona	13	16	48
University of Georgia	21	49	48
Binghamton University, State University of New York	19	45	50
Maxwell School, Syracuse University	21	30	50
University of California, Santa Barbara	23	34	50
University of Florida	11	23	50
TOTAL	1597	2348	

Table S3. Number of Students in the Study by Gender and by University.
