# Supplementary material for: Does Encouragement Matter in Improving Gender Imbalances in Technical Fields? Evidence from a Randomized Controlled Trial
Source: PLoS One. 2016 Apr 20;11(4):e0151714. doi: 10.1371/journal.pone.0151714 (PMC4838300; doi:10.1371/journal.pone.0151714)
Supplement: S4 Table — Data on the treatment status of students, disaggregated by home department and gender. (PDF) [file pone.0151714.s004.pdf]

Treated/Control Subjects by Department

University	Treated Female	Control Female	Treated Male	Control Male	Dept. Rank
Harvard	34	34	51	50	1
Princeton	20	19	48	48	2
Stanford	24	24	23	22	3
University of Michigan	19	18	20	20	4
Yale University	26	26	33	33	4
University of California, Berkeley	26	27	38	38	6
Columbia University	39	38	49	50	7
MIT	16	16	24	24	8
University of California, San Diego	15	16	37	38	8
Duke	14	14	22	22	10
University of California, Los Angeles	25	24	34	34	10
University of Chicago	30	30	37	38	12
University of North Carolina, Chapel Hill	18	17	23	22	13
Washington University in St. Louis	6	6	12	12	13
New York University	14	13	28	27	15
Ohio State University	14	13	24	25	15
University of Rochester	8	7	17	17	15
University of Wisconsin-Madison	16	16	18	19	15
Cornell University	20	21	25	25	19
University of Minnesota, Twin Cities	17	17	24	24	19
Northwestern University	23	23	27	28	21
The University of Texas at Austin	22	23	39	38	21
University of California, Davis	9	10	21	21	23
University of Illinois at Urbana-Champaign	13	13	17	16	23
Emory University	10	9	9	9	25
Indiana University Bloomington	18	18	36	37	25
Texas A&M University, College Station	6	7	10	9	25
Penn State University	8	8	15	15	28
University of Maryland	9	10	14	13	28
University of Pennsylvania	12	12	18	18	28
University of Washington	24	24	21	22	28
Michigan State University	8	8	17	17	32
Rice	3	4	11	12	32
Stony Brook University	6	7	7	7	32
The University of Iowa	7	8	11	11	32
Notre Dame	17	17	26	27	36
The George Washington University	12	12	20	20	36
University of Virginia	15	14	20	19	36
Vanderbilt University	11	10	13	12	36
Florida State University	7	7	14	13	40
Georgetown University	29	29	31	31	40
Johns Hopkins University	3	2	4	3	40
University of California, Irvine	15	14	22	22	40
University of Pittsburgh	7	8	12	13	40
Brown University	12	12	14	15	45
Rutgers	26	26	20	20	45
University of Colorado, Boulder	13	12	19	20	45
University of Arizona	7	6	8	8	48
University of Georgia	11	10	25	24	48
Binghamton University, State University of New York	10	9	23	22	50
Maxwell School, Syracuse University	11	10	15	15	50
University of California, Santa Barbara	12	11	17	17	50
University of Florida	5	6	11	12	50
TOTAL	802	795	1174	1174	

Table S4. Treatment Status of Students in the Study by Gender and University.
