# Supplementary material for: Does Encouragement Matter in Improving Gender Imbalances in Technical Fields? Evidence from a Randomized Controlled Trial
Source: PLoS One. 2016 Apr 20;11(4):e0151714. doi: 10.1371/journal.pone.0151714 (PMC4838300; doi:10.1371/journal.pone.0151714)
Supplement: S1 Text — Text of the two emails used as the randomized interventions. (PDF) [file pone.0151714.s005.pdf]

Intervention Email Text

The first email intervention was sent on March 4, 2014. The second email intervention was sent on March 19, 2014. Both emails were sent from the personal account of the President of the Society for Political Methodology and were addressed in a personalized fashion.

First Email

```
Dear [Student's First Name],

I'm writing to you on behalf of the Society for Political Methodology
to encourage you to consider submitting a proposal for a poster
presentation at the 2014 Political Methodology Summer Meeting
(PolMeth).

To give you some background, the PolMeth summer meeting provides an
exciting opportunity for students at all stages of their graduate
careers. The conference is small and focused, bringing together
approximately 150 faculty and graduate students from across political
science. The highlight of the conference is the graduate student
poster session, in which student participants can expect to receive
high-quality, detailed feedback from leading scholars in the field.
Many successful political scientists attended PolMeth as graduate
students, and many credit this experience as being an important
catalyst to their careers.

The Society for Political Methodology encourages poster proposals from
all fields of political science, and especially welcomes submissions
from comparative politics, international relations, race and ethnic
politics, and gender and politics. We encourage submissions that focus
on applied substantive topics as well as on methodological
innovations.

I am also pleased to announce that the Society for Political
Methodology will be able to offer some graduate student participants
support for travel and lodging as well as conference registration
fees.

The application can be completed at: [Conference URL]
information about the conference can be found at: [Conference URL]
I hope you will consider submitting a poster proposal to the 2014
Summer Meeting.

If you should have any questions, please contact me at
[Quinn Email].
```

Please note that the deadline for applications is Friday, March 28 at 11:59 pm Eastern.

Sincerely,
Kevin Quinn

Kevin Quinn
Professor of Law
UC Berkeley School of Law
490 Simon #7200
University of California, Berkeley
Berkeley, CA 94720-7200
- - - - - - - - - - - - - - - - - - -
President
The Society for Political Methodology

Second Email

Dear [Student's First Name],

I wrote to you about two weeks ago to encourage you to consider submitting a proposal for a poster presentation at the 2014 Political Methodology Summer Meeting (PolMeth). If you have submitted a proposal, thank you. If you have not, I hope you will still consider submitting a proposal.

To again give you some background, the PolMeth conference provides an exciting opportunity for students at all stages of their graduate careers. The conference is small and focused, bringing together approximately 150 faculty and graduate students from across political science. The highlight of the conference is the graduate student poster session, in which student participants can expect to receive high-quality, detailed feedback from leading scholars in the field. Many successful political scientists attended PolMeth as graduate students, and many credit this experience as being an important catalyst to their careers.

The Society for Political Methodology encourages poster proposals from all fields of political science, and especially welcomes submissions from comparative politics, international relations, race and ethnic politics, and gender and politics. We encourage submissions that focus on applied substantive topics as well as on methodological innovations.

I am also pleased to announce that the Society for Political Methodology will be able to offer some graduate student participants support for travel and lodging as well as conference registration fees.

The application can be completed at:

[Conference URL]

and more information about the conference can be found at:

[Conference URL]

I hope you will consider submitting a poster proposal to the 2014 Summer Meeting. If you should have any questions, please contact me at [Quinn Email].

Please note that the deadline for applications is Friday, March 28 at 11:59 pm Eastern.

Sincerely,
Kevin Quinn

Kevin Quinn
Professor of Law
UC Berkeley School of Law
490 Simon #7200
University of California, Berkeley
Berkeley, CA 94720-7200
- - - - - - - - - - - - - - - - - - -
President
The Society for Political Methodology
