# Supplementary material for: Does Encouragement Matter in Improving Gender Imbalances in Technical Fields? Evidence from a Randomized Controlled Trial
Source: PLoS One. 2016 Apr 20;11(4):e0151714. doi: 10.1371/journal.pone.0151714 (PMC4838300; doi:10.1371/journal.pone.0151714)
Supplement: S2 Text — Examples of responses received to the two randomized interventions. (PDF) [file pone.0151714.s006.pdf]

Responses to the Email Intervention

Some students responded to one or both emails, some to express enthusiasm or frustration and others to ask questions. Here we present selected responses to the email intervention. These are intended to give a flavor of the kinds of responses and do not represent any kind of systematic analysis.

We did not respond to emails that simply wrote back with an acknowledgment or a stated expression that the student would/would not submit a proposal (for example Emails #1, 2, and 3, below). Following the protocol used in other encouragement-oriented studies, we did answer specific questions with consistently used stock phrases. An example of our responses follows Email #4, below. We have no reason to think that our subsequent (non-randomized) interactions with responding students affect the inferences presented in the main text. We do note, however, that a larger number of email responses appear to have come from men.

Email #1

```
Dear Professor Quinn,

Thank you for alerting my attention to the conference.
Unfortunately, I am unable to attend.

Sincerely,
[Student's Name]
```

Email #2

```
Hello Kevin,

I appreciate your invitation to participate in this summer's
Political Methodology Meeting. I am, however, a theorist
and one whose approach to scholarly research
is firmly rooted in the liberal arts tradition. Still,
many thanks for offering  me an opportunity to join this
year's conference. I hope you and the other
attendees enjoy your stay in Athens, Georgia.

Best,
[Student's Name]
```

Email #3

```
Hi Kevin,

Thank you for the email and all the great information.
```

I intend on submitting a proposal in the next couple weeks!

Best,
[Student's Name]

Email #4

Dear Kevin,

Thank you for the information about PolMeth. I'd be interested in submitting a proposal on a paper that I am currently working on, but I was wondering how polished should a proposal for Polmeth presentation be. I do have a baseline empirical design and some findings and also hope to have a better draft with more rigorous evidence by summer, but the current version definitely needs more time than just a few weeks.

Thanks for your time in advance!

Best,
[Student's Name]

Response to Email #4

Dear [Student's Name],

I'm glad to hear of your interest in the conference.

It's hard for me to say whether your paper is sufficiently polished to present. I suggest you talk to your dissertation advisor about this. He or she should be able to give you good advice. Good luck.

Best,
KQ
